# Children's recognition of slapstick humor is linked to their Theory of Mind

**DOI:** 10.3389/fcogn.2024.1369638

**Published:** 2024-05-22

**Authors:** Ebru Ger, Moritz M. Daum, Mirella Manfredi

**Affiliations:** ^1^Department of Psychology, University of Bern, Bern, Switzerland; ^2^Department of Psychology, University of Zurich, Zürich, Switzerland; ^3^Jacobs Center for Productive Youth Development, University of Zurich, Zürich, Switzerland

**Keywords:** humor recognition, emotion recognition, kindergarten children, Theory of Mind, slapstick

## Abstract

Humor is an important component of children's learning and development. Yet, the cognitive mechanisms that underlie humor recognition in children have not been well-researched. In this pre-registered study, we asked whether (1) 4- to 5-year-old children recognize and categorize a misfortunate situation as funny only if the victims show a funny bewildered face (slapstick humor), and not a painful or angry expression, (2) this ability increases with age, (3) it is associated with children's Theory of Mind (ToM) abilities, (4) it is related to the ability to recognize facial emotional expressions. In an online experiment platform, children (*N* = 61, *M*_age_ = 53 months) were asked to point to the funny picture between a funny and an affective picture. Then, children were asked to point to the happy, sad, fearful, or angry face among four faces displaying these emotions. Children's ToM was assessed using the Children's Social Understanding Scale (CSUS), which was filled out online by parents. Results showed that from the earliest age onward, the predicted probability of humor recognition exceeded the chance level. Only ToM but not age was a significant predictor. Children with higher ToM scores showed better humor recognition. We found no evidence for a relation between children's humor recognition and their recognition of any emotion (happy, sad, fearful, or angry). Our findings suggest that 4–5-year-old children recognize facial emotional expressions and slapstick humor, although these abilities seem unrelated. Instead, children's understanding of mental states appears to play a role in their recognition of slapstick humor.

## 1 Introduction

Humor is defined as the faculty of perceiving and expressing or appreciating what is amusing or comical (Stein, 1980) and is universal (Jiang et al., 2019). Humor is important not only for adults as a social tool but also as a crucial part of children's learning, development, and social interactions (Krogh, 1985; McGhee, 2019; Recchia and Loizou, 2019; Jackson et al., 2021).

McGhee's (1979) cognitive-stage theory is the most recognized reference for understanding humor development, reflecting four specific development phases tightly connected to children's cognitive development. In Stage 1 (18–20 months), children find incongruous actions toward objects humorous. Yet, in his later contributions, he proposes the display of humor, even in the first year of life, centered around adults' unusual behaviors (McGhee, 2002). In Stage 2 (20–24 months), children find incongruous labeling of objects and events humorous. In Stage 3 (2–7 years), their humor becomes more complex due to the sophistication of symbolic play and the mental representation of familiar objects. Therefore, their humor usually consists of conceptual incongruity, such as assimilating objects (e.g., using a shoe as a telephone). They appreciate slapstick and cartoons. As language skills become refined, deliberately mislabeling actions or objects becomes part of their humor. Around age five, children begin to laugh and repeat jokes they have heard without really understanding them (the “pre-riddle” period). Finally, in Stage 4 (7–11 years), children recognize the different forms of ambiguity in language (phonological, semantic, syntactic, etc.) and begin to understand and appreciate the concept of double meaning.

Previous studies on the development of humor suggest that the ability to comprehend and appreciate humor is tightly interrelated to the development of other cognitive skills, among which Theory of mind (ToM) can be counted. ToM entails the understanding that others have mental states, that is, expectations, intentions, desires, knowledge, emotions, beliefs, and other inner experiences, which guide their behaviors (Wellman et al., 2001). In humans, ToM seems to be an innate potential capacity that necessitates social and other types of experience over several years for its complete development (Perner et al., 1994). The commonly accepted trajectory follows a path from understanding intentional and goal-directed agency in infancy (around 9 months) to understanding the more complex mental states in childhood (Rakoczy, 2022). Age 4 is considered revolutionary as meta-representational ToM becomes full-fledged. This is the time when children typically pass false-belief tasks (Wellman et al., 2001). The development continues into adulthood with the refinement of meta-representation, including understanding complex and subtle emotions.

ToM abilities may help the viewers recognize the underlying social and emotional context, understand the motives of those involved, and appreciate the humorous or playful intent behind the incongruity. Hence, ToM may allow individuals to go beyond the initial recognition of incongruity and delve into the social and emotional aspects, such as understanding the perspectives of the victims, that contribute to the resolution of incongruity. Previous research on adults has suggested that brain activation of a specific brain network in response to humorous information might be associated with ToM abilities (Gallagher et al., 2000; Bartolo et al., 2006; Kohn et al., 2011). Furthermore, humor has been used to assess ToM in adults (Aykan and Nalçaci, 2018). However, the relationship between different types of humor and ToM is not yet clear (Samson, 2012). Several studies have shown a connection between ToM and language development, as the latter constitutes a facilitating element for thinking about human behavior (Astington and Jenkins, 1999; De Villiers and de Villiers, 2014). Attardo (1997) suggests that to understand humor, a certain number of cognitive strategies are needed to deal with an unexpected or an incongruous element. Multiple studies have shown that people with cognitive deficits or brain lesions show impaired mentalization and humor understanding, suggesting that to understand humor properly, ToM skills are needed (Schnell, 2012). However, the potential link between ToM and humor recognition in children remains to be investigated.

In this work, we focused on slapstick humor, which is particularly appreciated by children between 2 and 7 years of age (McGhee, 1979; Shultz, 1996; Acuff and Reiher, 1997). It is characterized by physical and nonverbal comedy involving misfortunate situations, clownish behavior, and anthropomorphism. Writers and philosophers tried to comprehend why people usually laugh at the misfortunes of others and suggested that it may be because these misfortunes assert the person's superiority on the background of others' shortcomings (Hobbes, 1948; Freud, 1961). Although this form of humor has attracted people's interest in culture and art, very few studies have investigated the cognitive mechanisms underlying slapstick humor.

Recently, researchers sought to identify the key perceptive element that triggers a humorous reaction in adult observers while viewing scenes of slapstick comedy (Manfredi et al., 2014, 2017). The prototypical expression of bewilderment was hypothesized to act as a trigger for the reaction of amusement when observing a misfortunate situation, such as someone falling over. Confirming this hypothesis, adults viewing slapstick situations, including individuals with a bewildered and funny facial expression (Comic condition), showed larger perceptual electrophysiological responses (a posterior N170 and an anterior N220) compared to those including individuals with a painful or angry facial expression (Affective condition) or with their face not visible (No face condition, Manfredi et al., 2014). The N170 component is sensitive to facial expression (Hinojosa et al., 2015) and the N220 component is sensitive to stimuli containing conflicting features, such as the fearful expression of a victim in a non-life-threatening context (Luck and Kappenman, 2013). They may, therefore, represent an index of the first identification of a comic element (facial expression) during the observation of comic misfortunate situations. In addition, the No Face condition evoked a typical N400 response, an electrophysiological potential, which is an index of semantic analysis (Kutas and Federmeier, 2011). This response might reflect a difficulty in comprehending the nature of the situation observed, suggesting that facial expression may be the key element that enables humor recognition in ambiguous contexts. Moreover, tDCS stimulation on the superior temporal gyrus (STG), a brain region involved in the recognition of facial expressions, led to faster categorization of comic situations in adults, corroborating the evidence that the facial expression of the victims may act as a trigger for humor recognition (Manfredi et al., 2017). It is still an open question whether children recognizing slapstick humor would rely on recognizing facial emotional expressions.

The most widely accepted cognitive theory of humor processing is the two-stage theory (Suls, 1972; Vrticka et al., 2013; see Wyer and Collins, 1992 for a version expanded to three stages). The two-stage theory proposes that humor is composed of detecting an incongruity that violates expectations in the first stage and the resolution of that incongruity, which brings about amusement in the second stage. Misfortune situations often involve unexpected or incongruent events, where something unexpected or undesired happens to someone. The incongruity lies in the unexpected nature of the situation. The victim's bewildered facial expression serves as a crucial signal of incongruity. It contrasts with the expectation that the person would react with anger or pain in such a situation. After detecting this incongruity, viewers may undergo a cognitive shift, where they reinterpret the misfortune situation in a way that reduces tension and introduces humor. The bewilderment on the victim's face may signal that the situation is not as serious as it initially appears. The absence of an angry or painful expression contributes to the perception of harmlessness. Viewers recognize that the misfortune, while unexpected, would not result in severe harm or negative consequences. The bewildered facial expression enhances the incongruity, making the situation more unexpected and, in turn, more humorous.

In light of the reviewed evidence and previous work on adults, this study asked whether (1) 4–5-year-old children recognize and categorize a misfortunate situation as funny only if the victims show a funny bewildered face (slapstick humor), and not a painful or angry expression, (2) this ability increases with age, (3) it is associated with children's Theory of Mind (ToM) abilities, (4) it is related to the ability to recognize facial emotional expressions. To this aim, we tested children on a facial emotion recognition and a humor recognition task and asked parents to fill out a questionnaire assessing their child's ToM. We have chosen this age group because previous studies have observed that the most common forms of humor at this age include violations, exaggerations, or distortions of objects and actions properties (McGhee, 2002), and humor develops with increasing age (Bariaud, 1989). Furthermore, as mentioned above, the ability to recognize facial expressions develops non-linearly until about age six (Widen, 2013). Finally, there are indications in the literature that males and females differ in certain aspects of humor processing (Kohn et al., 2011; Chan, 2016), and boys show more hostile humor, while girls show more verbal humor (Groch, 1974; Bergen, 1989). Moreover, sibling interactions provide a good context for humorous exchanges (Paine et al., 2021). Therefore, we ran preliminary tests to check whether gender and number of siblings in our sample influenced their humor recognition.

## 2 Method

### 2.1 Participants

The final sample consisted of *N* = 61 kindergarteners (32 female) between the ages of 49 and 58 months (*M* = 53, *SD* = 2.6). The exclusion criteria for participating in the study were having a developmental disorder, impaired sight, being born preterm, or being bi- or multilingual. The parents accompanying the child and completing the questionnaire were 92% (*N* = 56) mothers and 8% (*N* = 5) fathers. The second caregiver was reported to be 90% (*N* = 55) fathers, 8% (*N* = 5) mothers, and 2% (*N* = 1) grandparents. Eighty-five percent (*N* = 52) of the responding parent and 100% of the second caregiver possessed a university equivalent degree. Eighty-seven percent (*N* = 53) of the children had siblings, 81% (N = 43) of those had only one, and 19% (N = 10) had two.

The participants were recruited from Zurich, Switzerland, via e-mail, using the participant database of the Research Unit at the University of Zurich. After completing the task and upon entering their address, participants were sent a certificate and a small present worth approximately CHF 5,- by post as a thank you. All procedures were approved by the local ethics committee and performed following the ethical standards of the 1964 Helsinki Declaration and its later amendments. All parents gave informed consent before data collection.

### 2.2 Materials, stimuli, and design

The service provider Gorilla Experiment Builder (www.gorilla.sc) was used to create and host our experiment online (Anwyl-Irvine et al., 2020). We also collected gaze data during the humor recognition task using the eye-tracking functionality embedded in Gorilla. However, because the quality of the obtained gaze data was deficient and many children could not successfully do the calibration for the eye tracking part, we do not report these data.

We assessed three main variables: ToM, emotion recognition, and humor recognition. We used the Children's Social Understanding Scale (CSUS) to measure the children's ToM (Tahiroglu et al., 2014). We used self-developed forced-choice tasks to measure facial emotion recognition and humor recognition. In the facial emotion recognition task, we used face stimuli with emotional expressions (2 females, 2 males) taken from the Radboud Faces Database (RaFD; Langner et al., 2010). The task consisted of 24 trials presented randomly. In each trial, four pictures of actors (2 male, 2 female) were presented, each displaying one of four emotions (happiness, sadness, fear, and anger). Actors in the target picture were pseudo-counterbalanced in a way that each actor depicted with each of the four emotions was the target at least once. In the humor recognition task, images depicting people involved in misfortunate situations (e.g., falling, spilling a drink) were grouped into two categories based on the facial expression of the victim: angry or painful (Affective) and bewildered and funny (Comic). The stimuli were derived from those used by Manfredi et al. (2014, 2017). The people depicted in each pair of images were balanced for gender, age, and body parts involved. The task consisted of training and 32 test trials presented in four blocks of six trials and a last block of eight trials. The presentation of the blocks and the trials within blocks were randomized. Between each block, an elephant image appeared where children were instructed to press the space key on the keyboard.

### 2.3 Procedure

Before the experiment, parents filled out the CSUS, the informed consent form, and a questionnaire on parents' working status (i.e., percentage of employment) and education (i.e., the highest level of education attained). The participating children were tested online while one parent was present. The parents were asked not to change their child's responses and correctly enter them, regardless of whether they think the answer is correct. Before the test began for each task, parents and children watched a pre-recorded video in which the experimenter gave instructions for the task. The order of the two tasks was counterbalanced. Children were also presented with a short age-appropriate cartoon movie between the tasks as a pause.

**Figure 1:**
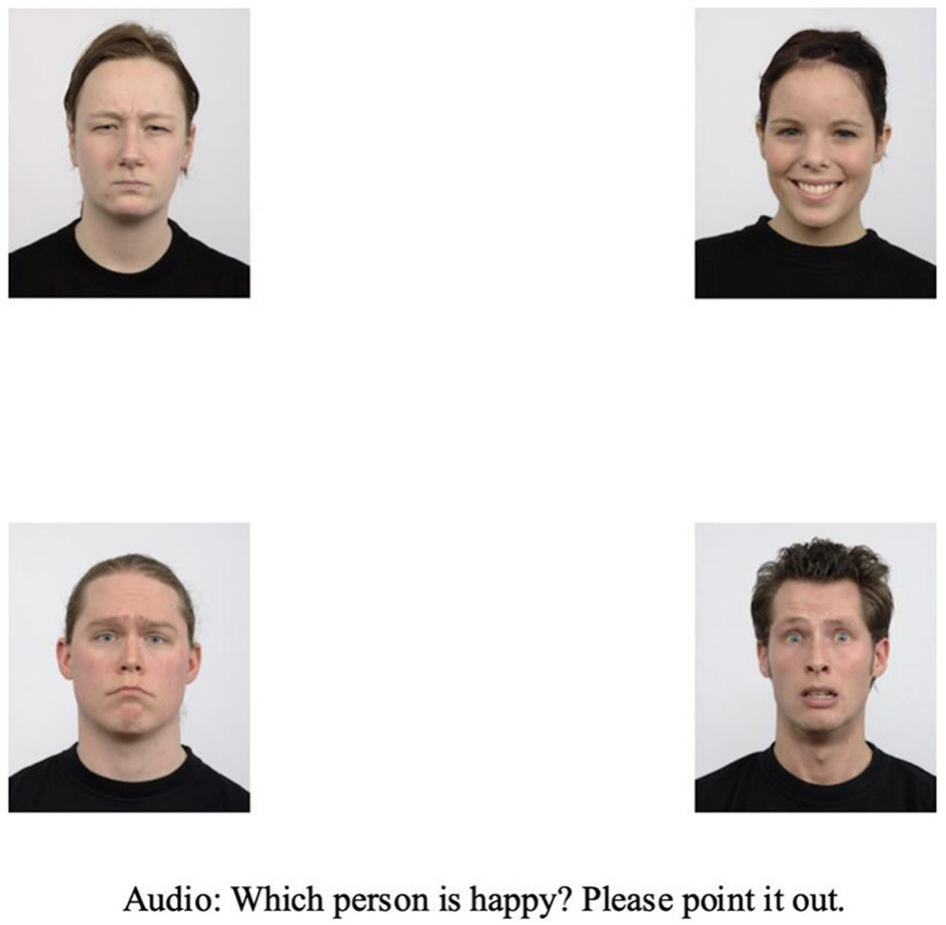
An example trial of the emotion recognition task. The depicted faces (taken from RaFD, Langner et al., 2010) are numbers 22, 37, 28, and 71, respectively, for the top left, top right, bottom left, and bottom right.

In the facial emotion recognition task, children saw four pictures of actors, each displaying one of 4 emotions (happiness, sadness, anger, and fear), and were asked by a pre-recorded auditory prompt, “Which is the happy/sad/fearful/angry face? Please point out” (Figure 1). Parents were asked to register which picture their child pointed to by clicking with their curser on the picture the child selected. A trial ended as soon as the parent clicked on the picture. Between trials, a fixation cross appeared for 250 ms with a 100 ms pause before and after. For each trial, children were given a score of 1 if they pointed to the correct picture and 0 otherwise.

**Figure 2:**
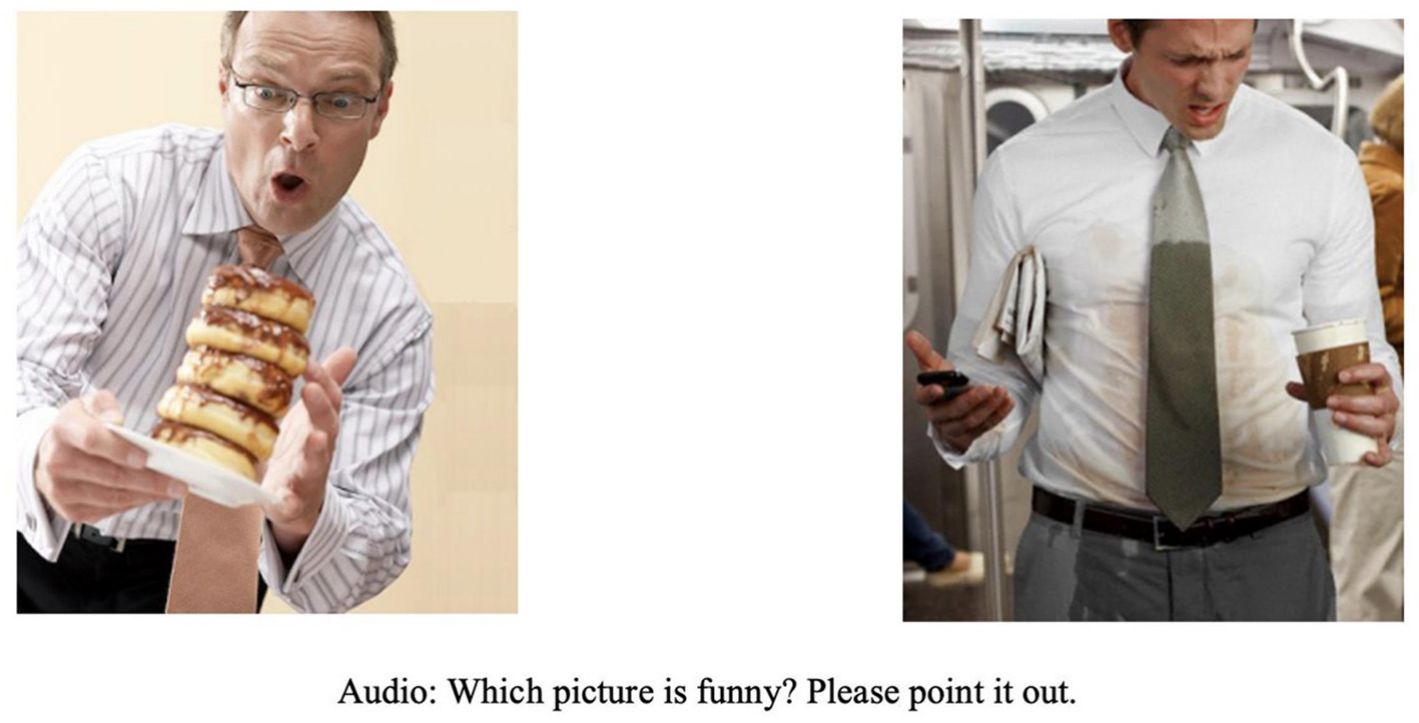
An example trial of the humor recognition task. The pictures, all copyrights free, were taken from Manfredi et al. (2014, 2017).

In the humor recognition task, parents were asked to register which picture their child pointed to by pressing the left or right key on the keyboard, corresponding to whether the child selected the image on the left or right. Children first received a practice trial where they saw a funny (comic) and an affective picture side by side for 2,000 ms, preceded and followed by a 250 ms fixation cross presented in the middle of the screen. Then they heard an audio asking them to point to the funny picture (“Which picture is funny? Please, point it out.”). After 5,000 ms, a script appeared on the bottom of the screen, prompting parents to press the left or the right key. The practice trial aimed to familiarize children with pointing at the screen when they heard the prompt question. In the test trials, children first saw a fixation cross in the middle of the screen for 250 ms. Then, pairs of stimuli (one comic and one affective) were presented for 6,000 ms at the center of the screen. Children saw the fixation cross again for 250 ms and afterward heard the auditory prompt while viewing the stimuli (Figure 2). The stimuli remained on the screen until the parent pressed one of the keys, upon which the trial ended. For each trial, children were given a score of 1 if they pointed to the comic picture and 0 otherwise.

### 2.4 Data analysis

We first ran a preliminary multiple linear regression to test whether gender (female as reference level) and number of siblings played a role in children's humor recognition score (percentage of funny pictures chosen). Next, we conducted a generalized linear mixed model with humor recognition as a binary dependent variable per trial (0: incorrect, 1: correct), age, ToM score, and emotion recognition score (percentage of correct emotion chosen) of happy, sad, fearful, and angry emotions as continuous fixed effects, and participant and trial as random intercepts. Age, ToM scores, and emotion recognition scores for each emotion were centered and scaled (i.e., transformed to z-scores) to facilitate model fit and interpretation. We expected individuals to vary in their baseline humor recognition; however, we did not have a theoretically based expectation that the relationship between predictor variables and humor recognition should differ between individuals. That is why we only added a random intercept and not a random slope by participant. We included trial also only as random intercept because the model with random slopes for trial within participant failed to converge. The R notation for the final model is reported below. We visualized and inspected the residuals and the distribution of the random effects from this model and did not observe any indications of non-normality.

glmer(humor_recognition ~ Age_scaled + ToM_scaled + happy_scaled + sad_scaled + fearful_scaled + angry_scaled + (1|Participant) + (1|trial), family = binomial(link = ‘logit')).

To answer our first question, whether children recognize and categorize a misfortunate situation as funny only if the victims show a funny bewildered face (slapstick humor) and not a painful or angry expression, we tested whether and at what age the lower bound of the 95% confidence interval for the predicted probability exceeds the chance level (50% because there are two response options). To answer our second question of whether humor recognition increases with age, we tested whether age was a significant predictor. To answer our third question, whether humor recognition is associated with children's Theory of Mind (ToM) abilities, we tested whether ToM was a significant predictor. To answer our fourth question, whether humor recognition is related to the ability to recognize facial emotional expressions, we assessed whether the recognition score of any emotion was a significant predictor. We pre-registered to run another mixed logistic model with an interaction term between emotion recognition score and type of emotion as a fixed effect instead of the recognition of each emotion as separate predictors. However, we deviated from this based on the feedback we received during the peer review process.

## 3 Results

This study was pre-registered on the Open Science Framework (OSF). The pre-registration, the anonymized data, and the analysis script are available at: https://osf.io/s2qth/. Data were analyzed using R [version 4.1.3] (R Core Team, 2020) and the packages “lme4” (Bates et al., 2015), “sjPlot” (Lüdecke, 2023), “rstatix” (Kassambara, 2023) and “emmeans” (Lenth et al., 2022).

**Figure 3:**
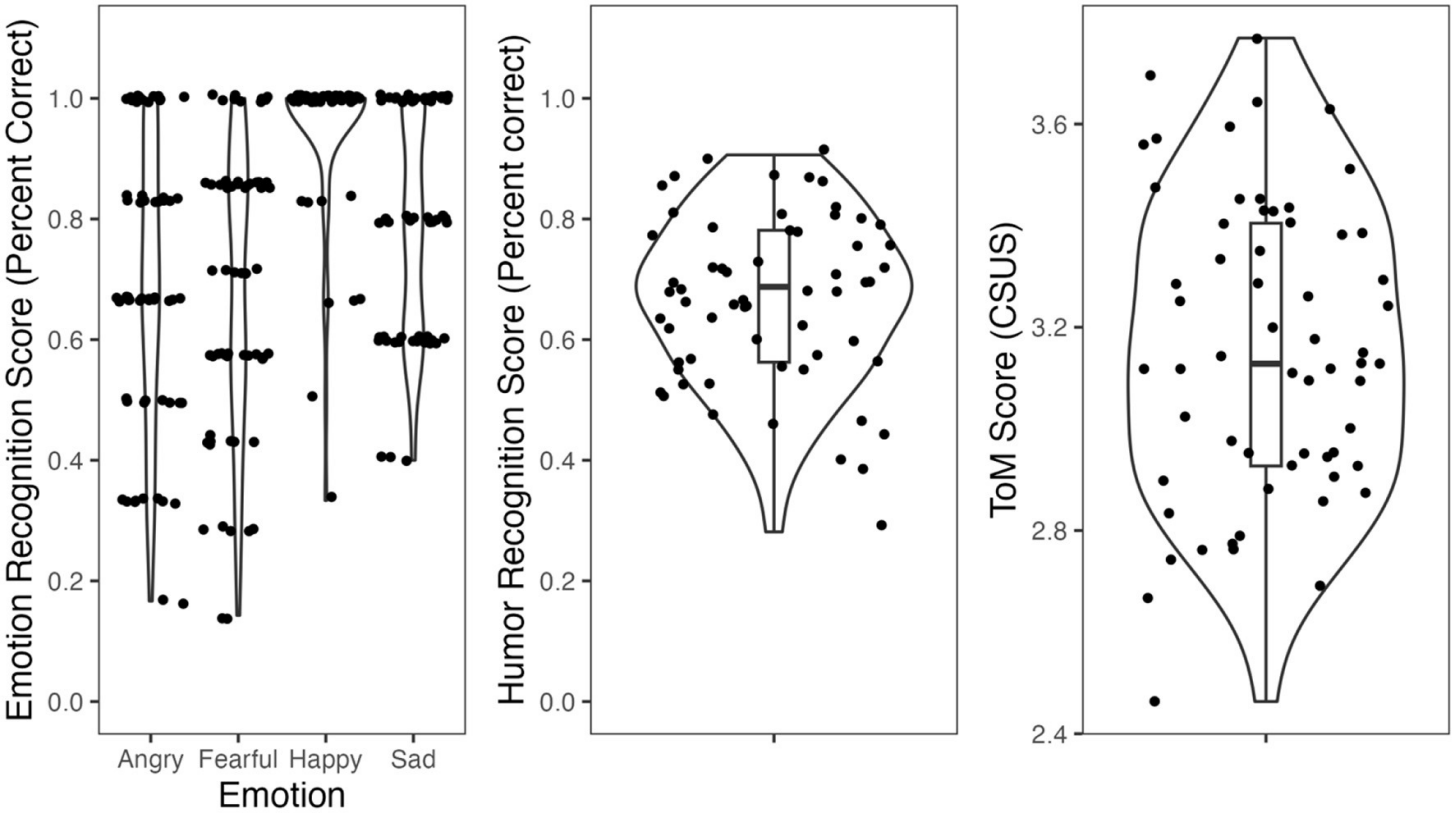
Descriptive visualizations of variables.

A total of 61 children completed all 36 trials and were included in the analyses. The results of the preliminary analysis did not reveal a significant influence by either gender (Beta = 0.02, *SE* = 0.04, *t* = 0.55, *p* = 0.588) or number of siblings (Beta = − 0.01, *SE* = 0.05, *t* = − 0.13, *p* = 0.895); therefore, they were not further considered in the analyses. The descriptive statistics can be found in Table 1, and visualizations can be found in Figure 3. Please note that the percentages are transformed to a scale of 0 to 1.

**Table 1:** Descriptive statistics.

**Variable**	***N***	**Mean**	**SD**	**Min**	**Max**
Humor recognition	61	0.67	0.14	0.28	0.91
ToM score	61	3.2	0.29	2.5	3.8
Emotion recognition	61	0.78	0.14	0.38	1
Angry	61	0.69	0.24	0.17	1
Fearful	61	0.68	0.24	0.14	1
Happy	61	0.95	0.13	0.33	1
Sad	61	0.79	0.19	0.4	1

**Figure 4:**
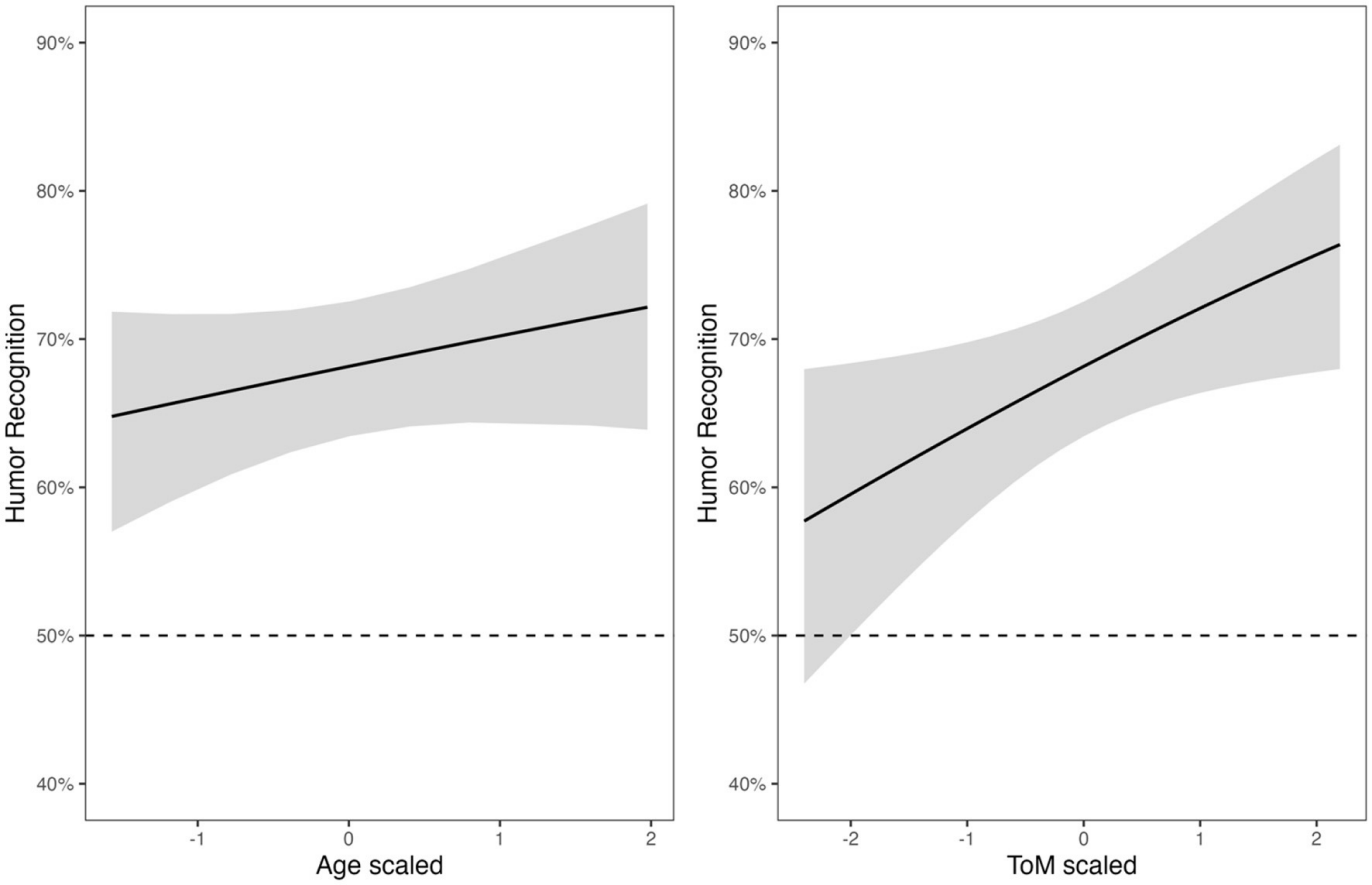
Predicted probability of correct humor recognition by age and ToM. The dashed lines on the graphs represent the chance level of 50%. For age scaled, 0 represents the mean age of 53 months, and each unit is the SD of 2.6 months. For ToM scaled, 0 represents the mean CSUS score of 3.2, and each unit is the SD of 0.29.

Regarding our first research question and hypotheses, the results of the mixed model showed that from the earliest age tested onward, the predicted probability of humor recognition (i.e., rating only the faces with funny bewilderment as funny) exceeded the chance level of 50% as can be seen in Figure 4, where 95% CI exceeds 50%. Although both age and ToM showed an increasing trend, only ToM but not age was a significant predictor (Table 2). Children with a higher ToM score showed better humor recognition. Specifically, one *SD* increase in the ToM score was associated with 1.21 times increase in the odds of correctly recognizing a funny picture.

**Table 2:** Summary of model predicting correct humor recognition by age, ToM, and emotion recognition.

**Predictors**	***Odds ratios***	***CI***	***p***
(Intercept)	2.17	0.562–7.64	0.227
Age scaled	1.10	0.94–1.29	0.239
ToM scaled	1.21	1.02–1.42	**0.025**
Happy	0.67	0.18–2.46	0.542
Sad	1.20	0.45–3.21	0.716
Fearful	0.73	0.35–1.51	0.415
Angry	1.89	0.91–3.92	0.088
**Random effects**			
σ^2^	3.29		
τ_00 Participant_	0.24		
ICC	0.16		
N _Participant_	61		
N _trial_	32		
Observations	1,952		
Marginal R^2^/Conditional R^2^	0.018/0.124		

This model summary is generated using the tab_model function from the sjPlot package in R.

The CI is calculated as the exponential of the Estimate ±1.96^*^SE. P-values in bold are <0.05.

**Figure 5:**
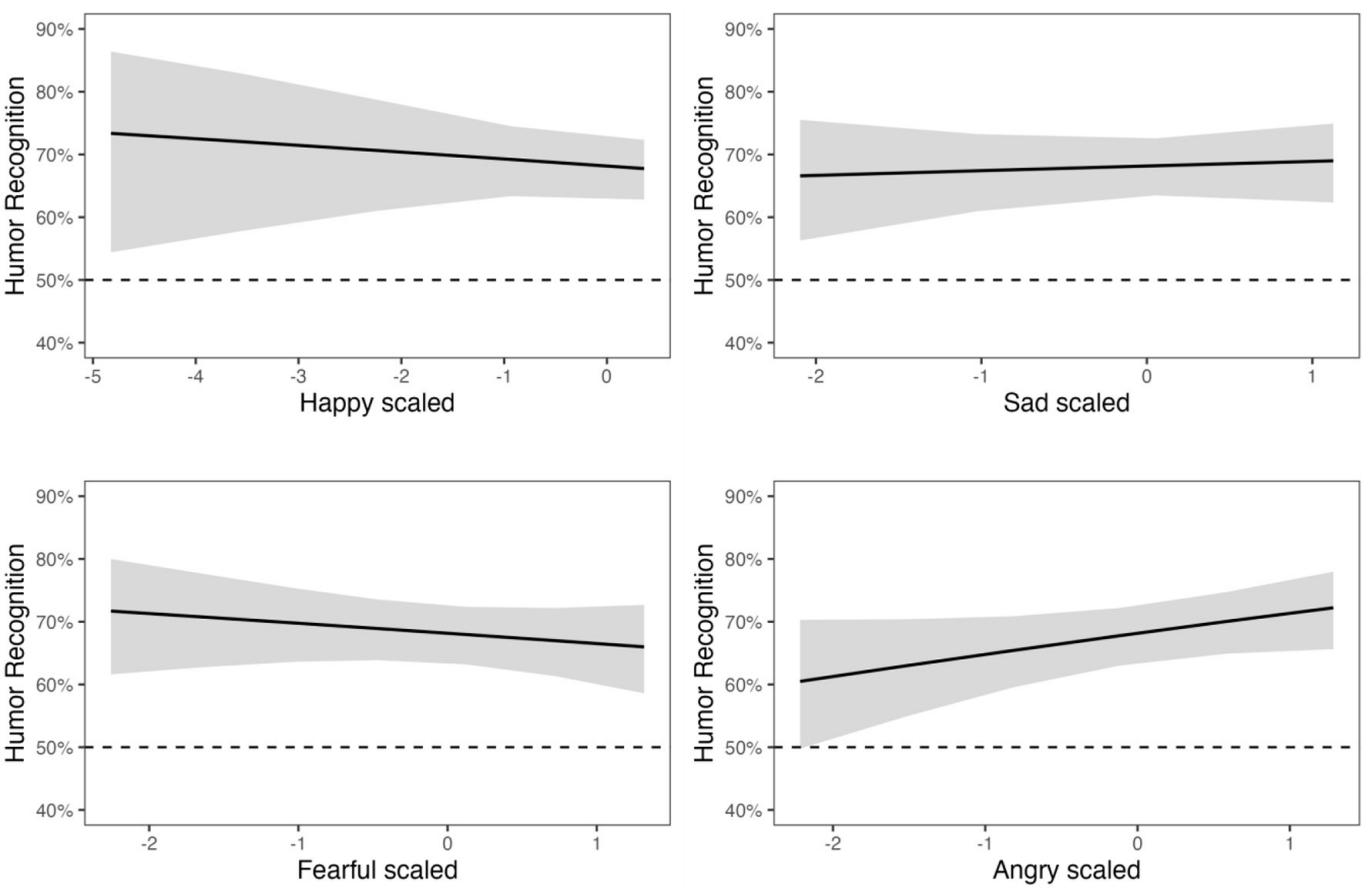
Predicted probability of correct humor recognition by recognition score of each emotion. The dashed lines on the graphs represent the chance level of 50%.

Regarding our second research question, we did not find the recognition score of any emotion to be a significant predictor of humor recognition (Table 2). That is, we found no evidence that children's humor recognition was related to their recognition of any emotion (happy, sad, fearful, or angry). However, the predicted probability of humor recognition exceeded the chance level of 50% for the total range of recognition scores for all the emotions, as seen in Figure 5, where the ribbon of 95% exceeds 50%.

## 4 Discussion

In this study, we first examined whether 4–5-year-old children recognize slapstick humor, that is, categorize a misfortunate situation as funny only if the victims show a funny bewildered but not a painful or angry facial expression. Next, we examined whether this ability changes as a function of children's age, recognition of facial emotional expressions, and Theory of Mind (ToM). The results showed that children recognized slapstick humor at a level above chance from the beginning of the age window we tested, and age was not a significant predictor of this recognition. Although the descriptive statistics showed that children recognized emotions well above chance, this was not found to be a significant predictor, whereas ToM was. Children with better ToM abilities were also better at identifying slapstick humor.

Four- to five-year-old children in our study reliably categorized a misfortune situation only when the victim showed a funny but not angry or painful facial expression. The extent to which they did so was not found to be significantly predicted by the extent of their recognition of facial emotional expressions (happy, sad, fearful, or angry). Yet the emotion closest to approaching significance was anger. This is not surprising because the humor recognition task required identifying a misfortune situation not as funny when the victim's face displays anger. Because children's recognition of emotions on average was also already at a considerably good level, it might no longer have had predictive power. Although emotion recognition did not predict humor recognition, as the main difference between the comic and affective picture alternatives in the humor recognition task was the facial expression of the victims, it is still highly likely that children's identification of the comic pictures relied on the facial expressions. Perhaps the recognition of emotional expressions in faces, not presented as standalone, but in the relevant misfortune context is key in identifying the humorousness. Previous studies with adults using neurophysiological measures found more robust evidence that indeed the recognition of facial expression determines the identification of misfortune as funny or not funny (Manfredi et al., 2014, 2017). Our study used behavioral measures and a correlational design as a first step in revealing potential links between emotion and humor recognition. It is necessary to address this question in children more directly, using neurophysiological and behavioral measures. A very recent study from our group with 4–5-year-old children using EEG found a significantly larger N170 component for funny compared to affective pictures, suggesting fast and automatic processing of the facial expression, providing the first insights in this direction (Manfredi et al., 2024).

Children's humor production and comprehension develop from 2 to about 11 years of age (Bariaud, 1989). We expected the recognition of slapstick humor to also develop within our tested age range of 4 to 5 because, around this age, children produce and appreciate humor that concerns conceptual incongruity before children proceed to a more complex level of humor that features double meanings or ambiguity (McGhee, 2002). Although we found an increasing trend with age, this was not significant. Hence, the recognition of slapstick humor may develop across a broader age range, not just between 4 and 5 years. The developmental progression of humor recognition might also vary within the 4–5-year age range. Some children may be at the beginning of grasping slapstick humor, while others may be more advanced. This variation could contribute to a lack of significance in the overall trend, and individual differences in cognitive and social-emotional development, such as ToM, may explain more variance in humor than age.

Children's recognition of slapstick was positively associated with their parent-reported ToM abilities. This aligns with previous research in adults showing associations between ToM and humor processing (Gallagher et al., 2000; Bartolo et al., 2006; Uekermann et al., 2008; Kohn et al., 2011). The current finding suggests that a similar link could already exist in childhood. Children may use their understanding of others' mental states, particularly emotional states, to recognize the underlying social and emotional context of the misfortune situation and appreciate the humorous or playful intent behind the incongruity. Let us take the example of seeing a picture of a person slipping and about to fall with a bewildered facial expression. Capitalizing on their ToM abilities, children may interpret this situation within the broader social and emotional context and understand that slipping and falling in this context is not a real threat or harm but rather a playful or humorous scenario. Their understanding of others' mental states may also help them appreciate the playful or humorous intent behind the incongruity, that is, recognize that the person's bewildered facial expression might be part of the comedic effect, signaling surprise or confusion rather than distress.

Humor is a powerful tool that fuels the development of physical, cognitive, language, and psychosocial skills (Robinson, 1991; Franzini, 2002). It may, for instance, facilitate children's social relations by supporting their communication and sense of mastery and joy. It may help the child to learn what is or is not socially appropriate. For instance, through practicing slapstick humor, children may learn not to laugh at a misfortune when the victim shows indications of pain. Humor can also boost children's creativity and reduce stress (Shade, 1996; Boyle and Stack, 2014). Our finding that 4–5-year-old children can appreciate slapstick humor as one form of humor implies that they may benefit from this ability in other social and cognitive domains and for their wellbeing. Humor is increasingly recognized as a valuable tool in therapy with children (Bernet, 1993; Southam, 2005; Berg et al., 2009). A valuable implication of the current findings is that slapstick humor may be utilized for such therapeutic purposes with 4–5-year-old children. Given that we found ToM to explain part of the variance in slapstick humor recognition, targeting ToM and humor understanding in concert could be a promising avenue in developing interventions for promoting young children's psychological wellbeing and sociocognitive development.

### 4.1 Limitations and future directions

We would like to note some limitations of the present study. First, we had children answer in a forced-choice yes-no fashion, which of two pictures was funny. That brings with it the limitation that children may have chosen a picture to be funny although they did not think it was funny *per se* but it was the funnier alternative. We also did not assess behavioral outputs of amusement, such as whether the children smiled or laughed upon seeing or choosing the funny picture. Nevertheless, our aim was instead to determine whether children can differentiate a humorous misfortune based on the victim's facial expression, and not how funny they find slapstick humor. Therefore, a forced-choice response already sufficed to serve this purpose.

Second, we only tested the basic emotions of happiness, sadness, fear, and anger. One reason we did not find that emotion recognition was predictive of humor recognition could be the particular emotions tested in the current study. It would be useful to add other emotions, such as surprise and disgust, in future research, which would allow us to better differentiate the development of emotion recognition skills in pre-school children.

Third, we assessed ToM only through a parent-reported questionnaire (i.e., CSUS). Although the CSUS is a widely used questionnaire to assess ToM, that is valid and reliable (Tahiroglu et al., 2014), it is common to combine it with a behavioral measure of ToM. Although parent reports are valuable sources of information, they are still subjective and may be influenced by parental biases or perceptions. Future studies are necessary to corroborate the current findings using more direct behavioral assessments of children's ToM abilities.

Fourth, because of technical difficulties and low data quality, we could not use the eye-tracking data, which would have been an important implicit measure for our humor and emotion recognition tasks.

Finally, a follow-up experiment including a more extensive emotion recognition test and a control condition (i.e., the presentation of a disconcerted expression in a non-comic condition) will be beneficial in clarifying some aspects of this work.

### 4.2 Conclusion

Our findings suggest that 4–5-year-old children recognize slapstick humor, namely, identify misfortunes as funny only if the victim shows a bewildered but not an angry or painful facial expression. This recognition does not appear to be related to the recognition of facial emotional expressions (happy, sad, fearful, angry) *per se*, but perhaps the recognition of these expressions in the context of the misfortune situation. Moreover, children's recognition of slapstick humor is linked with their parent-reported ToM abilities. This suggests that children may be using their understanding of others' mental states, in particular emotional states, to resolve the incongruity by recognizing the harmless and playful, therefore humorous nature of a slapstick situation.

## Data availability statement

The datasets presented in this study can be found in online repositories. The names of the repository/repositories and accession number(s) can be found below: https://osf.io/wbgqd/.

## Ethics statement

The studies involving humans were approved by the Ethics Committee of the Faculty of Arts and Social Sciences at the University of Zurich. The studies were conducted in accordance with the local legislation and institutional requirements. Written informed consent for participation in this study was provided by the participants' legal guardians/next of kin.

## Author contributions

EG: Conceptualization, Data curation, Formal analysis, Investigation, Methodology, Project administration, Resources, Supervision, Visualization, Writing – original draft, Writing – review & editing. MD: Funding acquisition, Writing – review & editing. MM: Conceptualization, Investigation, Methodology, Project administration, Resources, Supervision, Writing – review & editing.
